# Effect of Suvorexant vs Placebo on Total Daytime Sleep Hours in Shift Workers: A Randomized Clinical Trial

**DOI:** 10.1001/jamanetworkopen.2020.6614

**Published:** 2020-06-02

**Authors:** Jamie M. Zeitzer, Daniel S. Joyce, Amanda McBean, Yvonne L. Quevedo, Beatriz Hernandez, Jon-Erik Holty

**Affiliations:** 1 Stanford Center for Sleep Sciences And Medicine, Department of Psychiatry and Behavioral Sciences, Stanford University, Stanford, California; 2 Mental Illness Research, Education, and Clinical Center, VA Palo Alto Health Care System, Palo Alto, California; 3 Pulmonary, Critical Care, and Sleep Medicine Section, VA Palo Alto Health Care System, Palo Alto, California; 4 Division of Pulmonary and Critical Care Medicine, Department of Medicine, Stanford University, Stanford, California

## Abstract

**Importance:**

Many shift workers have difficulty sleeping during the daytime owing to an inappropriately timed circadian drive for wakefulness.

**Objective:**

To determine whether a dual hypocretin receptor antagonist would enable shift workers to have more daytime sleep.

**Design, Setting, and Participants:**

This double-blind, placebo-controlled randomized clinical trial included 2 weeks of baseline data and 3 weeks of intervention data, from March 2016 to December 2018. Individuals were recruited through poster advertisements in the broader San Francisco Bay area in California. From an initial voluntary recruitment cohort of 38 shift workers, 19 individuals with self-reported difficulty sleeping during the daytime following night work shift were included. Data were analyzed from Janaury to March 2019.

**Interventions:**

1 week of 10 mg suvorexant or placebo, titrated upward to 20 mg suvorexant or placebo for 2 additional weeks.

**Main Outcomes and Measures:**

Objective (ie, actigraphy) and subjective (ie, sleep logs) measures of sleep.

**Results:**

Among 19 participants who completed the study (mean [SD] age, 37.7 [11.1] years; 13 [68%] men), 8 participants (42%) were assigned to the suvorexant group and 11 participants (58%) were assigned to the placebo group. Compared with individuals in the placebo group, individuals in the suvorexant group increased their objective total sleep time by a mean (SE) of 1.04 (0.53) hours (*P* = .05) at the end of 1 week of 10-mg doses and by 2.16 (0.75) hours (*P* = .004) by the end of the 2 weeks of 20-mg doses. Subjective sleep was similarly improved as, compared with the placebo group, individuals in the suvorexant group increased their subjective total sleep time by a mean (SE) of 2.08 (0.47) hours (*P* < .001) at the end of 1 week of 10-mg doses and by 2.97 (0.56) hours (*P* < .001) by the end of the 2 weeks of 20-mg doses. Physician ratings of daytime sleep aligned with these measures, as there was no change in the placebo group and a much improved change in the suvorexant group. No adverse events were reported in the suvorexant group.

**Conclusions and Relevance:**

This pilot study found that the use of a dual hypocretin receptor antagonist in shift workers under real-world conditions resulted in more than 2 extra hours of daytime sleep per episode. Future research should confirm this pilot finding in a larger sample size and examine whether, over the long term, use of this medication has a concomitant improvement in medical and psychiatric health as well as workplace performance and safety.

**Trial Registration:**

ClinicalTrials.gov Identifier: NCT02491788

## Key Points

**Question:**

Can a dual hypocretin receptor antagonist increase daytime sleep in shift workers?

**Findings:**

In this pilot randomized clinical trial including 19 shift workers, daytime sleep quantity measured by actigraphy increased by a mean (SD) of 2.16 (0.75) hours, a significant improvement as compared with placebo.

**Meaning:**

These findings suggest that use of a dual hypocretin receptor antagonist may increase daytime sleep in shift workers; larger trials comparing suvorexant with current standard therapies should be explored.

## Teaser

This randomized clinical trial examines suvorexant vs placebo as a daytime sleep aid among shift workers.

## Introduction

Many shift workers have difficulty sleeping. The cardinal feature of shift work disorder is an inability to maintain sleep for extended periods of time during the day, which is due to a misalignment of the internal circadian clock with sleep and wake behavior.^1^ In other words, shift workers attempt to sleep when their circadian clock is signaling for them to be awake. A variety of techniques have been used to treat shift work disorder, including timed light therapy, melatonin, and hypnotic medications, although these have been met with limited success.^2^

It has been proposed that the circadian signal for wake promotion is mediated by hypocretin-1.^3^ Hypocretin-1 and -2 (also known as *orexins*) are excitatory neuropeptides produced by a diffuse group of neurons in the lateral hypothalamus,^4^ the loss of which results in the sleep disorder narcolepsy.^5^ Individuals with narcolepsy have a deficit in the circadian wake-promoting drive,^6^ and the pattern of hypocretin-1 in the lumbar cerebrospinal fluid of humans^7^ and cisterna magna of the diurnal, wake-consolidating squirrel monkeys^8^ are consistent with the hypothesis that, in a wake-consolidating species, such as humans, hypocretin-1 is, in part, a physiologic representation of the circadian wake drive.^3^ As circadian wake-promotion is the presumptive factor preventing adequate daytime sleep in shift workers, we hypothesized that blocking hypocretin-1 using the dual hypocretin receptor antagonist suvorexant would diminish the circadian wake-promoting drive and be permissive of increased sleep occurring during the day in shift workers.

## Methods

This randomized clinical trial was approved by the Stanford University institutional review board, and all procedures conform with the principles laid out in the Declaration of Helsinki.^9^ All participants signed an informed consent form prior to any study testing. This study followed the Consolidated Standards of Reporting Trials (CONSORT) reporting guideline.

Between March 1, 2016, and October 4, 2018, we recruited shift workers from the San Francisco Bay area in California. To be enrolled, we required participants be aged 20 to 60 years and engaged in full-time shift work, defined as working for at least 6 hours between 8 pm and 8 am with no shifts longer than 12 hours, as well as working a minimum of 4 nights per week or 32 hours of night shifts per week. Participants needed to have a minimum of 3 months of shift work prior to enrollment and a concern of difficulty sleeping during the daytime. General health was assessed with an interview by a clinician, electrocardiograph, urinalysis, and blood tests (ie, basic metabolic panel, complete blood count, liver function test). Exclusion criteria included pregnancy (verified by urine pregnancy test) or planning to become pregnant, currently breastfeeding, having an inadequate opportunity (ie, <7 hours) for daytime sleep after working an overnight shift, being an extreme evening or morning type (as assessed by a reduced version of the Horne & Östberg Morningness-Eveningness Questionnaire^10^), evidence of elevated depressive symptoms (ie, score >27 on the Center for Epidemiologic Studies Depression Scale [CES-D]),^11^ use of sleep aids (including as needed or continuous use of prescription, nonprescription, and naturopathic pharmacotherapies) at any point during the study period, diagnosis or detection of sleep disordered breathing on home sleep testing, diagnosis of narcolepsy, diagnosis of restless legs syndrome or detection of such through questionnaire,^12^ intake of more than 600 mg of caffeine per night shift or use of prescription stimulant medication during night shift, rotational or irregular work shifts during the study, severe hepatic impairment, unstable or severe medical or psychiatric condition, or the use of digoxin or strong or moderate cytochrome P450 3A4 isozyme inhibitors or cytochrome P450 3A4 isozyme inducers for 6 months prior to or during the study. Information concerning exclusionary criteria was obtained through questionnaires. Race/ethnicity was self-assessed by participants. Baseline screening questionnaires were administered using Stanford University REDCap, a secure web-based data collection application.

Eligible participants were scheduled for 2 weeks of at-home, ad libitum sleep monitoring to define usual daytime sleep after night shifts (baseline). During baseline, participants wore a wrist actigraph (Motionlogger [Ambulatory Monitoring]), which objectively records arm movement and data that can be used to impute the amount of sleep and wake^13^ and completed daily sleep logs after both nighttime and daytime sleep.^14^ On 1 night during baseline, participants’ overnight breathing patterns and pulse oximetry were recorded with an ApneaLink sleep testing device (ResMed) to screen for sleep apnea, defined by an apnea-hypopnea index of more than 10, as calculated by ApneaLink software version 10.20.

Following 2 weeks of baseline data collection, participants were randomized in a 1:1 ratio to receive either suvorexant or placebo for 21 days. Assignment to condition was performed through randomization in blocks of 4, with equal numbers randomized to each condition. Neither the participant nor any study staff member who had contact with the participants knew of the allocation assignment. One study member (J.M.Z.) generated the randomization sequence using Random.org (Randomness and Integrity Services) and a second team member determined which randomized group would receive placebo; this team member also held the blind. During the first treatment week (ie, phase 1), participants were instructed to swallow 1 pill of either 10 mg suvorexant or matching placebo before each daytime sleep episode. We requested that a participant ingest the pill within 1 hour of an attempt to initiate daytime sleep. After the first week, participants returned to the laboratory to discuss the effect of the pill on their sleep with a single clinician (J.-E. H.). Participants who felt that the pill was inadequate (partially or fully) in improving their sleep were titrated upward and assigned to use 2 pills (ie, double-dose of placebo or 20 mg of suvorexant) for the next 2 weeks (ie, phase 2). Adherence with taking the pills was assessed using MEMS Caps adherence monitoring system (Aardex Group) to record pill bottle openings, the times of which were offloaded at laboratory visits. Besides our inclusion criteria, there were no further restrictions or recommendations on the timing of sleep or work. Actigraphy and sleep logs continued through 3 weeks during which a pill was taken before daytime sleep.

### Measurements

After each day and night sleep episode, participants completed a sleep log with information concerning the sleep episode, including timing; latency to sleep onset; frequency, timing, and number of awakenings; and subjective sleep quality (SSQ) on a 5-point Likert-like scale (with 1 indicating very poor and 5, very good).^14^ These data were used to calculate subjective values for total sleep time (TST), sleep onset latency (SOL), and SSQ. Data from these logs were also used to frame the time of getting into and out of bed, which were used in analyses of the actigraphy data. Actigraphy data were analyzed with ActionW version 2.7 (Ambulatory Monitoring Inc) using 0-crossing mode and the Cole-Kripke algorithm^15^ for imputing sleep and wake from activity data.^13^ Actigraphy-determined TST, our a priori primary outcome measure, was calculated for each daytime sleep episode. During phase 1 and 2 of the trial, only daytime sleep attempts that were preceded by a pill bottle opening were included in analyses. We had preplanned to examine nocturnal sleep on off-days but these data have not been analyzed.

At the initial visit, a board-certified sleep medicine physician (J.-E. H.) completed a Clinical Global Impressions–Severity (CGIS) assessment.^16^ The CGIS scale is a 7-point Likert-like scale that asks the question, “Considering your total clinical experience with this particular population, how ill is the patient at this time?” in reference to their daytime sleep. Scores range from 1 (ie, normal, not at all ill) to 7 (ie, among the most extremely ill patients). At the end of baseline (ie, before initiation of the pill), a CGI-Improvement (CGII) scale was completed by the same physician. The CGII is a 7-point Likert-like scale that asks the question, “Compared to the patient's condition at admission to the project [prior to medication initiation], this patient's condition is:” in reference to their daytime sleep. Scores range from 1 (ie, very much improved since the initiation of treatment) to 7 (ie, very much worse since the initiation of treatment). A CGII was also administered by the same physician after 1 week of pill administration (ie, the end of phase 1) and at the end of the study (ie, the end of Phase 2). Depression symptoms (as measured by CES-D) were also assessed before each of the 3 physician visits (ie, baseline, end of phase 1, end of phase 2). Full details on the study protocol are presented in Supplement 1.

### Statistical Analysis

Semicontinuous data (ie, daily sleep logs and actigraphy) were analyzed with linear mixed-effects models using the *LME4* package in R statistical software version 3.6.0 (R Project for Statistical Computing).^17^ Questionnaire data, including the CGII, obtained at discreet time points were analyzed with repeated measure analysis of variance (ANOVA) using OriginPro2017 data analysis and graphing software (OriginLab). Standardized mean differences were calculated to examine effect size differences in baseline data.^18^ Data on SSQ were converted from a categorical scale (ie, very poor, poor, fair, good, and very good) to an ordinal scale (ie, 0, 1, 2, 3, and 4) for statistical purposes. Data on SOL were log transformed prior to statistical analysis to correct for the log normal distribution of these data. *P* values were 2-sided, and the significance level was set at *P* < .05. Data were analyzed from January to September 2019.

Our study was originally powered from published data on the effect of suvorexant on nighttime sleep in healthy men.^19^ From these data, we conservatively estimated that the variance in the increase in total sleep time could be as much as 150% of the observed variance at the 10 mg dose of suvorexant (ie, 27.1 minutes), and set our α at 0.05 and power at 80%, giving us an ability to detect a 25-minute change in total sleep time with 19 participants in each group. Owing to difficulties in recruitment, this number was halved. Recruitment difficulties also precluded our collection of prespecified secondary outcomes that potential participants found too burdensome, including measures of alertness and caffeine use on the actual night shifts. Full details on the original statistical analysis plan are included in Supplement 1.

## Results

**Figure 1.**
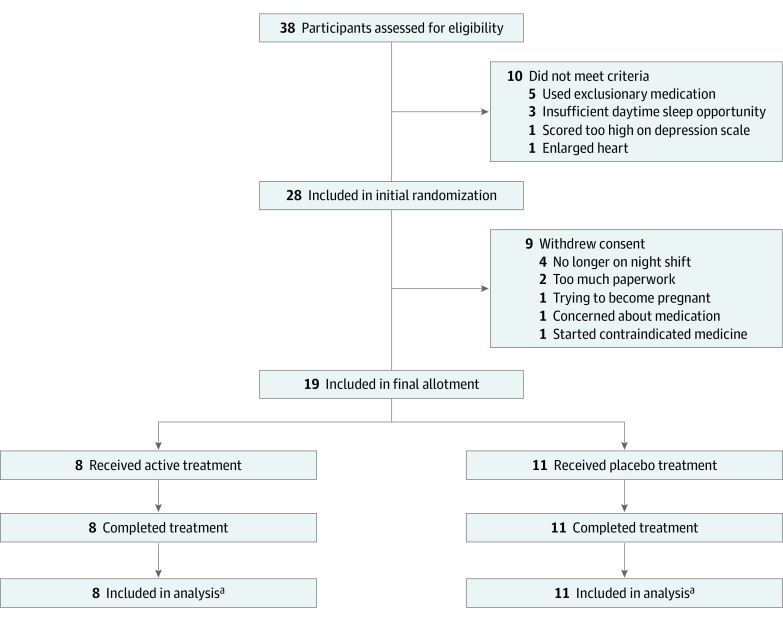
Diagram of Participant Flow Through Trial ^a^One participant in the suvorexant and 1 participant in the placebo treatment groups were excluded from actigraphy-based sleep analyses owing to data loss.

Of 38 individuals assessed for eligibility in the study (Figure 1), half were excluded or withdrew consent, and we obtained data from 19 participants (mean [SD] age, 37.7 [11.1] years; 13 [68%] men). There were no statistically significant differences between individuals who participated and those who did not in mean (SD) age (37.7 [11.1] years vs 43.9 [13.0] years; *t* test, *P* = .13), sex (13 men and 6 women vs 11 men and 7 women; Fisher Exact test, *P* = .74), race/ethnicity (10 white individuals and 9 individuals of other race/ethnicity vs 5 white individuals and 13 individuals of other race/ethnicity; Fisher Exact test, *P* = .18), or body mass index (calculated as weight in kilograms divided by height in meters squared) (mean [SD], 27.7 [4.21] vs 28.4 [6.47]; *t* test, *P* = .68) of those who were completed and those who did not. Among these, 8 participants (42%) were assigned to the suvorexant group and 11 participants (58%) were assigned to the placebo group. They were split unevenly owing to withdrawal after randomization but before study participation. Demographic characteristics were similar among individuals randomized to the placebo and active conditions (Table 1).

**Table 1.** Baseline Demographic Characteristics

Characteristic	Placebo (n = 11)	Suvorexant (n = 8)	*P* value
Age, mean (SD), y	35.1 (9.3)	41.4 (12.9)	.23
Men, No. (%)	8 (73)	5 (63)	.99
White race, No. (%)	5 (45)	5 (63)	.65
BMI, mean (SD)	26.9 (4.4)	28.8 (4.0)	.68

Abbreviation: BMI, body mass index (calculated as weight in kilograms divided by height in meters squared). Continuous data are presented as mean (SD).

### Baseline Sleep Comparisons

The placebo and suvorexant groups had similar daytime sleep characteristics during the 2-week baseline (Table 1). Both groups had approximately 4 to 5 hours of sleep per daytime sleep episode and had poor or fair subjective sleep quality. The groups differed in the clinician CGIS scale, on which individuals in the placebo group had a median (IQR) score of 4 (3-4) and individuals in the suvorexant group had a median (IQR) score of 5 (4-5) (Mann-Whitney *U*, *P* = .04).

### Phase 1 and 2

All individuals had at least 1 week of 10 mg suvorexant or placebo dosing prior to daytime sleep attempts during phase 1. At the end of this week, all individuals in the suvorexant group decided to increase their dose to 20 mg and 9 of 11 participants in placebo group decided to increase their dose for the following 2 weeks during phase 2. Of 2 participants in the placebo group who decided not to increase their dose, 1 felt that a contemporaneous medical condition (ie, facial swelling) may have been due to the drug and did not want to further increase their dose; the other felt that the drug was affecting their evening exercise (ie, running) and did not want to further increase their dose.

### Subjective TST

**Figure 2.**
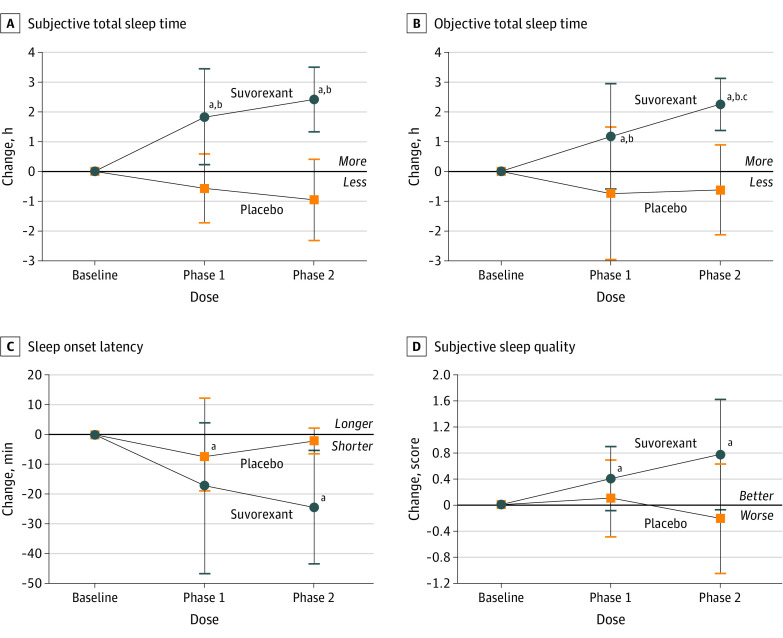
Change in Sleep Characteristics From Baseline to Phase 1 (10 mg) to Phase 2 (20 mg) ^a^Statistically significantly difference vs baseline. ^b^Statistically significantly difference vs placebo. ^c^Statistically significantly difference vs phase 1 (10 mg). Data on subjective total sleep time (TST) (A), sleep onset latency (C), and subjective sleep quality (SSQ) (D) were derived from patient-reported sleep logs; objective TST (B), from actigraphy. Participants receiving placebo are shown as open boxes; those receiving suvorexant are shown as closed circles. Points indicate mean, and whiskers, SD.

In the placebo group, subjective TST did not change across the study phases (Table 2; Figure 2A). In the suvorexant group, subjective TST increased from baseline in to phase 1 and to phase 2 (Table 2; Figure 2A). Between the placebo and suvorexant groups, the mean (SE) change from baseline to phase 1, was 2.08 (0.47) hours (*P* < .001), from baseline to phase 2 was 2.97 (0.56) hours (*P* < .001), and from phase 1 to phase 2 was 1.11 (0.59) hours (*P* = .06).

**Table 2.** Sleep Characteristics During Each Study Phase

Measure	Mean (SD)					
	Baseline		Phase 1 (10 mg)		Phase 2 (20 mg)	
	Placebo	Suvorexant	Placebo	Suvorexant	Placebo	Suvorexant
Episodes of daytime sleep, No.	8.5 (3.2)	8.1 (2.5)	5.0 (3.5)	4.0 (2.5)	5.6 (1.6)	5.5 (3.7)
Subjective TST, h^a^	5.41 (1.67)	4.52 (1.71)	4.50 (1.36)	6.51 (1.34)	4.71 (1.35)	6.90 (1.79)
Objective TST, h^b^	4.96 (1.55)	4.20 (1.01)	4.69 (1.36)	5.37 (1.78)	4.53 (1.32)	6.38 (1.87)
Sleep latency onset min^a^	18.1 (2.3)	27.9 (2.0)	13.6 (2.0)	20.7 (1.9)	17.6 (2.2)	14.5 (3.2)
Subjective sleep quality score^a^	2.0 (0.7)	1.4 (0.7)	2.0 (0.7)	1.8 (0.7)	2.0 (0.8)	2.1 (0.3)

Abbreviation: TST, total sleep time.

^a^ Measured by patient-reported sleep logs.

^b^ Measured by actigraphy.

### Objective TST

In the placebo group, objective TST did not change across the study phases (Table 2; Figure 2B). In the suvorexant group, objective TST increased from baseline to phase 1 and to phase 2, and also between phase 1 and phase 2 (Table 2; Figure 2B). Between the placebo and suvorexant groups, the mean (SE) change from baseline to phase 1 was 1.04 (0.53) hours (*P* = .05), from baseline to phase 2 was 2.16 (0.75) hours (*P* = .004), and from phase 1 to phase 2 was 1.72 (0.70) hours (*P* = .01).

### Subjective SOL

Subjective SOL had a slight decrease in phase 1 in the placebo group and a slight decrease in phase 2 in the suvorexant group (Table 2; Figure 2C). The changes in subjective SOL were not different between placebo and suvorexant groups. Between the placebo and suvorexant groups, the mean (SE) change from baseline to phase 1 was −2 (13) minutes (*P* = .89), from baseline to phase 2 was −18 (12) minutes (*P* = .15), and from phase 1 to phase 2 was −18 (16) minutes (*P* = .25).

### SSQ

In the placebo group, SSQ did not change across the study phases (Table 2; Figure 2D). In the suvorexant group, SSQ improved from baseline in both phase 1 and phase 2 (Table 2; Figure 2D). Between the placebo and suvorexant groups, the mean (SE) change in SSQ score from baseline to phase 1 was 0.31 (0.24) (*P* = .20), from baseline to phase 2 was 0.40 (0.27) (*P* = .14), and from phase 1 to phase 2 was 0.64 (0.30) (*P* = .03).

### CGI

**Figure 3.**
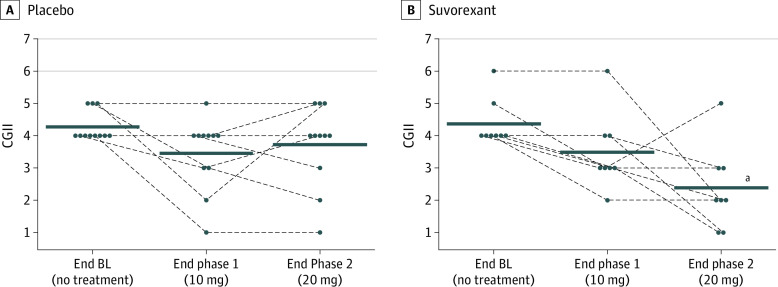
Change in Clinical Global Impressions Improvement (CGII) Scores Among Participants ^a^Statistically significant difference in placebo vs suvorexant and vs baseline is noted. Individual participants and their trajectories are shown as dots; group medians, horizontal black bars.

In the placebo group, CGIS started at a median (IQR) of 4 (3-4). Scores for CGII in the placebo group were at a median (IQR) of 4 (4-5) at end of baseline, 4 (3-4) at end of phase 1, and 4 (3-5) at end of phase 2 (Figure 3A). In the suvorexant group, CGIS started at a median (IQR) of 5 (4-5). Scores for CGII in the suvorexant group were at a median (IQR) of 4 (4-4.75) at the end of baseline, 3 (3-4) at the end of phase 1, and 2 (1.25-3) at the end of phase 2 (Figure 3B). The change over time was different between the placebo and suvorexant groups (2-way repeated measure ANOVA with Huynh-Feldt correction, *P* = .009), with Bonferroni post hoc testing indicating that there was a change in CGII scores between baseline and the end of phase 2 in the suvorexant group (*P* < .001).

### Adverse Events

Aside from 2 individuals in the placebo group, we received no other reports of adverse events. There were decreases in depressive symptoms, as measured by CES-D scores, between the end of phase 1 (mean [SD] change, −6.5 [4.3]) and phase 2 (mean [SD] change, −5.9 [2.2]) in the active group, but not in the placebo group (phase 1 mean [SD] change, −0.36 ± 4.4, phase 2 mean [SD] change, −0.90 [5.0]) (2-way repeated measure ANOVA with Huynh-Feldt correction, *P* = .002). However, post hoc Bonferroni analyses failed to confirm the difference in changes between the 2 groups at either time point.

## Discussion

The findings of this pilot randomized clinical trial indicate that in a field study of shift workers, the self-administration of a dual hypocretin antagonist prior to daytime sleep episodes can increase time asleep by more than 2 hours. This effect was observed in TST as determined by both objective measurement (ie, actigraphy) and subjective report (ie, sleep log). There was a marginal effect on SOL and SSQ. The improvements in self-reported and objectively measured sleep time were also supported by clinician rating scales that observed much improved symptoms by the end of the study in individuals who received suvorexant and no change in individuals who received placebo.

Most individuals who work night shifts do not remain on a permanent night schedule, as they are often active during the day and sleep at night on their off days. Such rapid, daily transitions between being night active and day active do not allow for proper synchronization of the circadian clock to the preferred behavioral schedule. Therefore, daytime sleep among shift workers often occurs at a time during which their circadian clock is signaling them to be awake.^20^ Daytime sleep initiation is usually possible in shift workers because they have spent a suitable amount of time awake, which builds up the homeostatic pressure to sleep and allows for the initiation of sleep.^20^ However, sleep length can be curtailed owing to the dissipation of homeostatic sleep pressure by sleep and the onset of the circadian wake drive. It has been previously demonstrated that hypocretin-1 is likely involved in the expression of this circadian wake drive.^8^ Thus, our a priori hypothesis was that the predominant improvement in daytime sleep following administration of a hypocretin dual receptor antagonist would be an increase in TST. Our data are consistent with this theory, as a large increase in TST was observed. We observed marginal effects for the 20 mg dose vs baselines (although not vs placebo) on SOL, which we did not expect to change, as well as SSQ. High variability in these measures may have contributed to a failure to observe statistical significance. Furthermore, we did not specifically recruit individuals who had difficulty initiating sleep during the daytime, so there may have been a boundary effect. If it were found to be reliable, the 20-minute decrease in SOL we observed would be clinically meaningful in individuals who had a difficult time initiating sleep during the daytime. The lack of statistically significant difference in SSQ is also not surprising, as there is often a dichotomy between objective measures of sleep and subjective measures of sleep quality.^21^

In this protocol, we opted to do an upward titration, from 10 mg to 20 mg of suvorexant, that was based on feedback from participants after the first week using the study drug (either 10 mg suvorexant or placebo). This follows the recommendations of the US Food and Drug Administration that calls for a 10-mg starting dose with upward titration not to exceed 20 mg daily for individuals in whom suvorexant is tolerated but not effective.^22^ All individuals who received 10 mg of suvorexant opted to increase their dose to 20 mg, despite the increase in TST that occurred after the first week of medication. The clinical observation (ie, CGII score) was that these individuals had subjectively experienced only minimal improvement after a week of treatment, which also agreed post hoc with their subjective sleep quality. The clinician-rated improvement was significant after the additional 2 weeks of 20 mg of drug treatment. We do not know whether continuation of 10 mg of drug treatment would have resulted in a similar increase in TST to what was observed in the 20 mg drug treatment or the improvement observed by the clinician, but it is a possibility that some of the increased effects of the 20 mg of drug were secondary to continued exposure to the study drug as opposed to the specific dose.

### Limitations

Our study has some limitations. Our study inclusion criteria were intentionally broad to capture the wide variety of individuals who work alternate shifts. We did specifically exclude individuals who had elevated depressive symptoms as measured by CES-D, although we still observed a decrease in depressive symptoms in the suvorexant group. We did not study individuals who worked both day and night shifts, although our results would be encouraging for these individuals as well. We also did not study individuals who lacked sufficient opportunity for sleep, often owing to family obligations in the afternoon (eg, picking up children from school or daycare in the afternoon). Given the long half-life of suvorexant (approximately 12 hours),^22^ it might be unwise to administer this drug to individuals who do not have a sufficiently extended time to sleep. We also did not examine whether there is long-term (ie, months to years) adaptation to the use of this drug. In this relatively small study, we did not observe any side effects directly attributable to the use of suvorexant. However, there are published adverse effects associated with use of this drug, most notably drowsiness, but also abnormal thoughts, behavior, and thinking, worsening of depression, and sleep paralysis or cataplexy-like symptoms.

## Conclusions

These findings suggest that administration of a dual hypocretin receptor antagonist prior to daytime sleep initiation in shift workers can substantially increase the amount of sleep they obtain. Given the difference in the mechanism of action of this antagonist and traditional sleep medications (eg, benzodiazepines and benzodiazepine receptor agonists), it will be important for future research to examine if there are differences in the amount and microarchitecture of daytime sleep in shift workers given these 2 classes of medications. Future research also will be necessary to determine if the increased sleep that is enabled by this medication is associated with an improvement in the negative medical (eg, hypertension) and work (eg, fatigue, performance) outcomes that are found in many shift workers.

## Notes

Supplement 1. **Trial Protocol and Statistical Analysis Plan**

Supplement 2. **Data Sharing Statement**
