# Supplementary material for: Effect of Suvorexant vs Placebo on Total Daytime Sleep Hours in Shift Workers: A Randomized Clinical Trial
Source: JAMA Netw Open. 2020 Jun 2;3(6):e206614. doi: 10.1001/jamanetworkopen.2020.6614 (PMC7267849; doi:10.1001/jamanetworkopen.2020.6614)
Supplement: Supplement 1. — Trial Protocol and Statistical Analysis Plan [file jamanetwopen-3-e206614-s001.pdf]

PROTOCOL
APPLICATION FORM
Human Subjects Research
Stanford University

Title : A double-blind, placebo-controlled, randomized, parallel design study to examine the effectiveness of 10 mg suvorexant in improving daytime sleep in shift workers

Approval Period: **Draft**

Protocol Director

Name		Degree (Program/year if student)		Position, e.g. Assistant Professor, Resident, etc.
Jamie Marc Zeitzer		PhD		Asst Professor
**Department**	**Mail Code**	**Phone**	**Fax**	**E-mail**
Psych/Sleep Medicine	5550	[redacted]		jzeitzer@stanford.edu
**CITI Training current**				Y

Admin Contact

Name		Degree (Program/year if student)		Position, e.g. Assistant Professor, Resident, etc.
Yvonne L Quevedo		BS		
**Department**	**Mail Code**	**Phone**	**Fax**	**E-mail**
Psychiatry and Behavioral Sciences	5550	[redacted]		Yvonne.Quevedo@va.gov
**CITI Training current**				Y

Investigator

Name		Degree (Program/year if student)		Position, e.g. Assistant Professor, Resident, etc.
**Department**	**Mail Code**	**Phone**	**Fax**	**E-mail**
**CITI Training current**				

Other Contact

Name		Degree (Program/year if student)		Position, e.g. Assistant Professor, Resident, etc.
**Department**	**Mail Code**	**Phone**	**Fax**	**E-mail**
**CITI Training current**				

Academic Sponsor

Name		Degree (Program/year if student)		Position, e.g. Assistant Professor, Resident, etc.
**Department**	**Mail Code**	**Phone**	**Fax**	**E-mail**
**CITI Training current**				

Other Personnel

Name		Degree (Program/year if student)		Position, e.g. Assistant Professor, Resident, etc.
Jon-Erik Holty		MD		Clinical Assistant Professor (Affiliated) [VAPAHCS]

Title : A double-blind, placebo-controlled, randomized, parallel design study to examine the effectiveness of 10 mg suvorexant in improving daytime sleep in shift workers

Approval Period: Draft

**Department**	**Mail Code**	**Phone**	**Fax**	**E-mail**
Medicine - Med/Pulmonary and Critical Care Medicine		[redacted]	[redacted]	Jholty@stanford.edu
**CITI Training current**				Y

**Name**		**Degree (Program/year if student)**		**Position, e.g. Assistant Professor, Resident, etc.**
Shannon Sullivan		MD		Clinical Assistant Professor
**Department**	**Mail Code**	**Phone**	**Fax**	**E-mail**
Psych/Sleep Medicine	5730	[redacted]		shannon.s.sullivan@stanford.edu
**CITI Training current**				Y

Participant Population(s) Checklist — Yes/No

- Children (under 18) — N
- Pregnant Women and Fetuses — N
- Neonates (0 - 28 days) — N
- Abortuses — N
- Impaired Decision Making Capacity — N
- Cancer Subjects — N
- Laboratory Personnel — N
- Healthy Volunteers — Y
- Students — N
- Employees — N
- Prisoners — N
- Other (i.e., any population that is not specified above) — N

Study Location(s) Checklist — Yes/No

- Stanford University
- Clinical & Translational Research Unit (CTRU)
- Stanford Hospital and Clinics
- Lucile Packard Children's Hospital (LPCH)
- VAPAHCS (Specify PI at VA) — Y
  Jamie Zeitzer
- Other (Click ADD to specify details)

General Checklist

Multi-site — Yes/No

Title : A double-blind, placebo-controlled, randomized, parallel design study to examine the effectiveness of 10 mg suvorexant in improving daytime sleep in shift workers

Approval Period: **Draft**

• Is this a multi-site study? A multi-site study is generally a study that involves one or more medical or research institutions in which one site takes a lead role.(e.g., multi-site clinical trial)	N

**Collaborating Institution(s)**	**Yes/No**
• Are there any collaborating institution(s)? A collaborating institution is generally an institution that collaborates equally on a research endeavor with one or more institutions.	N

**Cancer Institute**	**Yes/No**
• Cancer-Related Studies (studies with cancer endpoints), Cancer Subjects (e.g., clinical trials, behavior/prevention) or Cancer Specimens (e.g., blood, tissue, cells, body fluids with a scientific hypothesis stated in the protocol).	N

**Clinical Trials**	**Yes/No**
• Investigational drugs, biologics, reagents, or chemicals?	N
• Commercially available drugs, reagents, or other chemicals administered to subjects (even if they are not being studied)?	Y
• Investigational Device / Commercial Device used off-label?	N
• IDE Exempt Device (Commercial Device used according to label, Investigational In Vitro Device or Assay, or Consumer Preference/Modifications/Combinations of Approved Devices)	Y
• Will this study be registered on# clinicaltrials.gov? (See Stanford decision tree)	Y
• Is Stanford responsible for ClinicalTrials.gov registration? (See Stanford decision tree) NCT#	

**Tissues and Specimens**	**Yes/No**
• Human blood, cells, tissues, or body fluids (tissues)?	Y
• Tissues to be stored for future research projects?	N
• Tissues to be sent out of this institution as part of a research agreement? For guidelines, please see https://sites.stanford.edu/ico/mtas	N

**Biosafety (APB)**	**Yes/No**
• Are you submitting a recombinant DNA vector or Human Gene Transfer investigation using biological agents? If yes, please complete and attach the Gene Transfer Protocol Application Supplemental Questions to section 16 of the eProtocol application.	N
• Are you submitting a Human study using biohazardous/infectious agents? If yes, refer to the http://www.stanford.edu/dept/EHS/prod/researchlab/bio/index.html Administrative Panel on BioSafety website prior to performing studies.	N

• Are you submitting a Human study using samples from subjects that are known or likely to contain biohazardous/infectious agents? If yes, refer to the http://web.stanford.edu/dept/EHS/prod/researchlab/bio/index.html Administrative Panel on BioSafety website prior to performing studies.	N

**Human Embryos or Stem Cells**	**Yes/No**
• Human Embryos or Gametes?	N
• Human Stem Cells (including hESC, iPSC, cancer stem cells, progenitor cells)	N

**Veterans Affairs (VA)**	**Yes/No**
• The research recruits participants at the Veterans Affairs Palo Alto Health Care System(VAPAHCS).	N
• The research involves the use of VAPAHCS non-public information to identify or contact human research participants or prospective subjects or to use such data for research purposes.	N
• The research is sponsored (i.e., funded) by VAPAHCS.	N
• The research is conducted by or under the direction of any employee or agent of VAPAHCS (full-time, part-time, intermittent, consultant, without compensation (WOC), on-station fee-basis, on-station contract, or on-station sharing agreement basis) in connection with her/his VAPAHCS responsibilities.	Y
• The research is conducted using any property or facility of VAPAHCS.	Y

**Equipment**	**Yes/No**
• Use of Patient related equipment? If Yes, equipment must meet the standards established by Hospital Instrumentation and Electrical Safety Committee (650-725-5000)	N
• Medical equipment used for human patients/subjects also used on animals?	N
• Radioisotopes/radiation-producing machines, even if standard of care? http://www.stanford.edu/dept/EHS/prod/researchlab/radlaser/Human_use_guide.pdf More Info	N

**Payment**	**Yes/No**
• Subjects will be paid/reimbursed for participation? See payment considerations.	Y

**Funding**	**Yes/No**
• Training Grant?	N
• Program Project Grant?	N
• Federally Sponsored Project?	N
• Industry Sponsored Clinical Trial?	Y

Funding

Title : A double-blind, placebo-controlled, randomized, parallel design study to examine the effectiveness of 10 mg suvorexant in improving daytime sleep in shift workers

Approval Period: **Draft**

Funding - Grants/Contracts

Funding Administered By : PAVIR

SPO # (if available) :

Grant # (if available) :

Funded By (include pending) : Merck Pharmaceuticals

Principal Investigator : Jamie Zeitzer

Grant/Contract Title if different from Protocol Title :

Y For Federal projects, are contents of this protocol consistent with the Federal proposal?
N Is this a Multiple Project Protocol (MPP)?
N Is this protocol under a MPP?

Funding - Fellowships

Gift Funding

Dept. Funding

Other Funding

Resources :

a) Qualified staff.

Please state and justify the number and qualifications of your study staff.

Dr. Zeitzer is the PI of this study and has conducted studies on human sleep and circadian rhythms for the past 20 years, including clinical trial studies such as this. Drs. Holty and Sullivan are board-certified sleep physicians and have conducted clinical research projects with Dr. Zeitzer in the past. Ms. Quevedo has extensive experience working with clinical trials and been working on such studies with Dr. Zeitzer for the past several years.

b) Training.

Describe the training you will provide to ensure that all persons assisting with the research are informed about the protocol and their research-related duties and functions.

Dr. Zeitzer will ensure the proper conduct and training of all individuals associated with this project.

c) Facilities.

Please describe and justify.

This is primarily an out-patient study. Dr. Zeitzer has assigned office space at VAPAHCS that will be used for recruiting and interviewing participants. The Clinical Studies Unit at VAPAHCS will be used for participant clinical exams.

d) Sufficient time.

Explain whether you will have sufficient time to conduct and complete the research. Include how much time is required.

All staff will have sufficient time to conduct and complete the research. Ms. Quevedo, the study

coordinator, will spend the majority of her time on this study. Dr. Zeitzer will spend approximately 10% of his time supervising this study, Dr. Sullivan will spend approximately 5% of her time advising for this study, and Dr. Holty will spend approximately 5% of his time doing participant exams in this study.

e) Access to target population.

Explain and justify whether you will have access to a population that will allow recruitment of the required number of participants.

Healthy volunteers who do shift work will be recruited from the community. Given the prevalence of shift work in the catchment area of this study, we believe that there will be sufficient numbers of participants for recruitment.

f) Access to resources if needed as a consequence of the research.

State whether you have medical or psychological resources available that participants might require as a consequence of the research when applicable. Please describe these resources.

Drs. Sullivan and Holty will be on 24-hour call in case of medical issues arising during this study.

g) Lead Investigator or Coordinating Institution in Multi-site Study.

Please explain (i) your role in coordinating the studies, (ii) procedures for routine communication with other sites, (iii) documentation of routine communications with other sites, (iv) planned management of communication of adverse outcomes, unexpected problems involving risk to participants or others, protocol modifications or interim findings.

1. Purpose

a) In layperson's language state the purpose of the study in 3-5 sentences.

The purpose of this study is to determine if suvorexant, a drug that inhibits a wake-promoting (hypocretin/orexin) system in the brain, is able to improve day time sleep and night time alertness in individuals who work night shifts.

b) State what the Investigator(s) hope to learn from the study. Include an assessment of the importance of this new knowledge.

There are currently no acceptable therapies to treat day time sleep difficulties in individuals who work night shifts (Shift Work Sleep Disorder). Current pharmacotherapies involve use of benzodiazepines or other sleep medications that put a person into an unnatural state of sleep. The medication being tested in this study blocks a very specific wake-promoting system in the brain that will enable individuals to achieve normal sleep during the daytime.

c) Explain why human subjects must be used for this project. (i.e. purpose of study is to test efficacy of investigational device in individuals with specific condition; purpose of study is to examine specific behavioral traits in humans in classroom or other environment)

The physiology of the drug-targeted system (hypocretin/orexin) being examined in this study is distinct in primates as compared with rodents and other non-mammalian species. The medical condition being examined (Shift Work Sleep Disorder) is uniquely human.

2. Study Procedures

a) Please SUMMARIZE the research procedures, screening through closeout, which the human subject

will undergo. Refer to sections in the protocol attached in section 16, BUT do not copy the clinical protocol. Be clear on what is to be done for research and what is part of standard of care.

All procedures are done for research-purposes only.

Baseline visit (3 hours): Consent process followed by baseline assessments using questionnaires, blood draw, urinalysis, electrocardiogram, and physical exam
Week 1-2: Sleep will be monitored with a wrist-worn device that monitors movement as well as participant-completed logs. No changes in behavior.
Weeks 3-5: Sleep will be continued to monitored as during baseline. Subjects will also take a pill 30 minutes prior to day time sleep attempts. This pill will have either drug or placebo (randomized)
End of study visit (2 hour): Same procedures as baseline visit.

b) Explain how the above research procedures are the least risky that can be performed consistent with sound research design.

All procedures in this study were designed with the goal of minimizing exposure to risk balanced with gaining a greater understanding of whether this treatment would help the individuals who participate in this study and for whom there is no currently available FDA-approved therapy.

c) State if deception will be used. If so, provide the rationale and describe debriefing procedures. Since you will not be fully informing the participant in your consent process and form, complete an alteration of consent (in section 13). Submit a debriefing script (in section 16).

Deception will not be used.

d) State if audio or video recording will occur. Describe what will become of the recording after use, e.g., shown at scientific meetings, erased. Describe the final disposition of the recordings.

Audio and video recordings will not occur.

e) Describe alternative procedures or courses of treatment, if any, that might be advantageous to the participant. Describe potential risks and benefits associated with these. Any standard treatment that is being withheld must be disclosed in the consent process and form. (i.e. standard-of-care drug, different interventional procedure, no procedure or treatment, palliative care, other research studies).

Instead of participating, individuals who work night shifts can use any one of a number of behaviors or medications to help them sleep during the day time. None of the available pharmacotherapies induce natural sleep and the behavioral therapies have efficacy in only a subset of individuals.

f) Will it be possible to continue the more (most) appropriate therapy for the participant(s) after the conclusion of the study?

After conclusion of the study, participants would be able to contact their physicians to obtain a prescription for the medication used in this study.

g) Study Endpoint. What are the guidelines or end points by which you can evaluate the different treatments (i.e. study drug, device, procedure) during the study? If one proves to be clearly more effective than another (or others) during the course of a study, will the study be terminated before the projected total participant population has been enrolled? When will the study end if no important differences are detected?

The primary endpoint of this study is a change in total sleep time during day time sleep episodes. Given the blinding of the study and the relatively small population being studied, we do not intend to examine the primary outcome prior to study completion (38 total subjects).

3. Background

a) Describe past experimental and/or clinical findings leading to the formulation of the study.

The hypocretins (orexins) are a pair of neuropeptides produced by a small group of hypothalamic neurons

that are involved in the regulation of the wake state. As hypocretins are only consistently found in neural tissue and cerebrospinal fluid (CSF), examination of their normal physiologic role in humans has been limited. This is problematic in that the regulation of hypocretins appears to be highly species dependent and related to the manner by which the species consolidates the timing of their sleep and wake. Data from my laboratory, obtained in a series of experiments using the wake-consolidating squirrel monkey (Saimiri sciureus), indicate that hypocretin-1 is likely to be the physiologic representation of the mathematically modeled circadian alertness signal. This alerting signal increases in the afternoon and reaches a peak just before habitual bedtime. This signal offsets the increase in homeostatic sleep pressure that accumulates with time spent awake and allows for the consolidation of wake. Importantly, the circadian alertness signal is, as the name indicates, controlled by the central circadian pacemaker located in the hypothalamic suprachiasmatic nucleus. As such, its rise - and the rise of hypocretin-1 in wake-consolidating species such as humans - is determined by internal circadian clock time and is little affected by behavior such as sleep/wake patterns.

While this circadian alerting signal as mediated by hypocretins is indispensible for consolidation of sleep and wake (c.f., sleep and wake fragmentation in those with hypocretin-deficient narcolepsy), it is in direct conflict with obtaining sleep during the daytime, when the circadian alertness signal is working in direct opposition to obtaining sleep. One group of individuals who consistently try to sleep during the biological daytime, when hypocretin-1 concentrations are elevated and opposing sleep, are those who work shifts. According to a survey in 2004 by the U.S. Bureau of Labor Statistics, there are at least 15 million Americans who participate in shift work. While we have the scientific capacity to "flip" the circadian rhythms of individuals working at night and trying to sleep during the daytime, it is a slow process and cannot synchronize individuals who switch between working nights and being awake during the daytime on days off. This leaves two unpalatable options: get poor sleep that is both difficult to initiate and maintain, or take medications such as diphenhydramine (H1 receptor antagonist) or benzodiazepine receptor agonists that induce an artificial and not particularly restful sleep-like state.

Suvorexant provides an ideal solution for improving sleep in shift workers. Histamine H1 receptor antagonists and benzodiazepine receptor agonists force the brain into a state of non-wake. As a dual hypocretin receptor antagonist, suvorexant would suppress the potent circadian drive for wake, thereby creating a permissive state in which natural sleep would be allowed to occur.

b) Describe any animal experimentation and findings leading to the formulation of the study.

see 3a

4. Radioisotopes or Radiation Machines

a) List all standard of care procedures using ionizing radiation (radiation dose received by a subject that is considered part of their normal medical care). List all research procedures using ionizing radiation (procedures performed due to participation in this study that is not considered part of their normal medical care). List each potential procedure in the sequence that it would normally occur during the entire study. More Info

Identify Week/Month of study	Name of Exam	Identify if SOC or Research

b) For research radioisotope projects, provide the following radiation-related information:

Identify the radionuclide(s) and chemical form(s).

For the typical subject, provide the total number of times the radioisotope and activity will be administered (mCi) and the route of administration.

If not FDA approved provide dosimetry information and reference the source documents (package insert, MIRD calculation, peer reviewed literature).

c) For research radiation machine projects, provide the following diagnostic procedures:

For well-established radiographic procedures describe the exam.

For the typical subject, identify the total number of times each will be performed on a single research subject.

For each radiographic procedure, provide the setup and technique sufficient to permit research subject dose modeling. The chief technologist can usually provide this information.

For radiographic procedures not well-established, provide FDA status of the machine, and information sufficient to permit research subject dose modeling.

d) For research radiation machine projects, provide the following therapeutic procedures:

For a well-established therapeutic procedure, identify the area treated, dose per fraction and number of fractions. State whether the therapeutic procedure is being performed as a normal part of clinical management for the research participants's medical condition or whether it is being performed because the research participant is participating in this project.

For a therapeutic procedure that is not well-established, provide FDA status of the machine, basis for dosimetry, area treated, dose per fraction and number of fractions.

5. Devices

a) Please list in the table below all Investigational Devices (including Commercial Devices used off-label) to be used on participants.

b) Please list in the table below all IDE Exempt Devices (Commercial Device used according to label, Investigational In Vitro Device or Assay, or Consumer Preference/Modifications/Combinations of Approved Devices) to be used on participants.

5. 1 Device Name : Actiwatch2

Describe the device to be used.

Gross motor activity monitor (similar to a FitBit)

Manufacturer Respironics

IDE Exemption

Y **This is a legally marketed device being used in accordance with its labeling.**

5. 2 Device Name : ApneaLink

Describe the device to be used.

This device monitors breathing (nasal cannula) and blood oxygenation and heart rate (finger pulse oximetry)

Manufacturer Resmed

IDE Exemption

Y **This is a legally marketed device being used in accordance with its labeling.**

6. Drugs, Reagents, or Chemicals

a) Please list in the table below all investigational drugs, reagents or chemicals to be administered to participants.

b) Please list in the table below all commercial drugs, reagents or chemicals to be administered to participants.

6. 1 Drug Name : Suvorexant

Source (i.e. Pharmacy, Sponsor, etc.) : Merck Pharmaceuticals

If not pre-mixed, where will the material be mixed and by whom:

Manufacturer : Merck Pharmaceuticals

IND# (if available) :

Dosage : 10 or 20 mg

Administration Route:

oral

IND Exemption

**N**	Is this new and different uses of this commercially available drug, reagent or chemical?
**Y**	Are all of these IND Statements true?

Investigational New Drug (IND) Regulations

The IND Regulations [21 CFR 312.2(b)] state that clinical investigation of a drug product is exempt from the requirements for an IND if all of the following apply:

- The Drug used in the investigations is lawfully marketed in the United States.
- The investigation is not intended to be reported to FDA in support of new indication for use or to support any other significant change in the labeling for the drug.
- The investigation is not intended to support a significant change in the advertising of the product.
- The investigation does not involve a route of administration or dosage level, use in a participant population, or other factor that significantly increases the risks (or decreases the acceptability of the risks) associated with the use of the drug product.
- The investigation is conducted in compliance with the requirements for IRB review and informed consent [21 CFR parts 56 and 50].
- The investigation is conducted in compliance with the requirements concerning the promotion and sale of drugs [21 CFR part 312.7], e.g., the drug may not be represented as safe or effective for the purposes for which it is under investigation, nor may it be commercially distributed or sold.

7. Medical Equipment for Human Subjects and Laboratory Animals

If medical equipment used for human patients/participants is also used on animals, describe such equipment and disinfection procedures.

n/a

8. Participant Population

a) **State the following: (i) the number of participants expected to be enrolled at Stanford-affiliated site(s); (ii)the total number of participants expected to enroll at all sites; (iii) the type of participants (i.e. students, patients with certain cancer, patients with certain cardiac condition) and the reasons for using such participants.**

(i) We anticipate that we will enroll 38 participants to completion. We estimate that this will require 50 participants beginning the study (i.e., at least signing a consent form).
(ii) This is a single-site investigation
(iii) This study will examine healthy individuals who work at night and have difficulty initiating or maintaining sleep during the day. We are specifically studying the effectiveness of this medication on this condition.

b) **State the age range, gender, and ethnic background of the participant population being recruited.**

Age: 20-60 years
Gender: all
Ethnicity: all with recruitment targeting the ethnicity distribution of individuals involved in night work in the catchment area

c) **State the number and rationale for involvement of potentially vulnerable subjects in the study (including children, pregnant women, economically and educationally disadvantaged, decisionally impaired, homeless people, employees and students). Specify the measures being taken to minimize the risks and the chance of harm to the potentially vulnerable subjects and the additional safeguards that have been included in the protocol to protect their rights and welfare.**

Vulnerable subjects will not be specifically recruited.

d) **If women, minorities, or children are not included, a clear compelling rationale must be provided (e.g., disease does not occur in children, drug or device would interfere with normal growth and development, etc.).**

Children are not included as they do not typically work night shifts and the effect of this drug on their developing brain is unknown.

e) **State the number, if any, of participants who are laboratory personnel, employees, and/or students. They should render the same written informed consent. If payment is allowed, they should also receive it. Please see Stanford University policy.**

We are not specifically targeting laboratory personnel, employees, or students, though they are not explicitly excluded. Should they be recruited, they will sign the same consent as all subjects and receive payment as allowed.

f) **State the number, if any, of participants who are healthy volunteers. Provide rationale for the inclusion of healthy volunteers in this study. Specify any risks to which participants may possibly be exposed. Specify the measures being taken to minimize the risks and the chance of harm to the volunteers and the additional safeguards that have been included in the protocol to protect their rights and welfare.**

All participants will be healthy volunteers. The main risk to which they are exposed, over the risks they normally encounter in their lives, is taking a prescription medication. There is a favorable side-effect profile with minimal risk reported in taking this medication. The medication being prescribed is being used to specifically treat a sleep disorder in these volunteers.

g) **How will you identify and recruit potential participants about the research study? (E.g., by: chart**

review; notified by treating physician; response to ad). All final or revised recruitment materials, flyers, etc. must be submitted to the IRB for review and approval before use. You may not contact potential participants prior to IRB approval. See Advertisements: Appropriate Language for Recruitment Material.

Participants will be recruited by flyers and online advertising.

h) Inclusion and Exclusion Criteria.

Identify inclusion criteria.

• Aged 20-60 (older individuals excluded due to altered sleep-related circadian signaling)
• Males and females
• Shift worker
  # Minimum of three months of prior shift work
  # Will work minimum of four nights per week or 32 hours of night shift per week during study
  # "Night work" defined as having at least six hours of work occurring between 8 PM and 8 AM and no longer than 12 hours on shift
• Presence of DSM-5 defined Circadian Rhythm Sleep-Wake Disorder: Shift Work Type
  # Insomnia (SE < 88%) during attempted daytime sleep or excessive sleepiness during nocturnal wake

Identify exclusion criteria.

• Currently or planning to become pregnant
• Currently breastfeeding
• Inadequate opportunity (<7 hours) for daytime sleep after shift work
• Use of sleep aids during the study period. Includes as needed or continuous use of prescription, non-prescription, and naturopathic pharmacotherapies
• Diagnosis or detection (during study) of sleep disordered breathing (AHI>10) on home sleep testing; referral to clinical sleep program will be offered
• Diagnosis of narcolepsy
• Restless Legs Syndrome
• >600 mg caffeine intake per night shift or use of prescription stimulant medication during night shift
• Rotational or irregular work shifts during study
• Use of digoxin for six months prior to or during study
• Use of strong (e.g., etoconazole, itraconazole, posaconazole, clarithromycin, nefazodone, ritonavir, saquinavir, nelfinavir, indinavir, boceprevir, telaprevir, telithromycin, conivaptan) or moderate (e.g., amprenavir, aprepitant, atazanavir, ciprofloxacin, diltiazem, erythromycin, fluconazole, fosamprenavir, grapefruit juice, imatinib, verapamil) CYP3A inhibitors or CYP3A inducers (e.g., rifampin, carbamazepine, phenytoin) for six months prior to or during study
• Severe hepatic impairment
• Unstable or severe medical or psychiatric condition

i) Describe your screening procedures, including how qualifying laboratory values will be obtained. If you are collecting personal health information prior to enrollment (e.g., telephone screening), please request a waiver of authorization for recruitment (in section 15).

Screening procedures will be done in a private room at VAPAHCS. Laboratory values will be obtained after a nurse draws blood and collects urine from the participant at the VAPAHCS Clinical Studies Unit.

j) Describe how you will be cognizant of other protocols in which participants might be enrolled. Please explain if participants will be enrolled in more than one study.

We will ask subjects during the recruitment and screening process whether they are enrolled in any other study. Participants enrolled in other studies will not be empaneled in this study.

k) Payment/reimbursement. Explain the amount and schedule of payment or reimbursement, if any, that will be paid for participation in the study. Substantiate that proposed payments are reasonable and commensurate with the expected contributions of participants and that they do not constitute undue pressure on participants to volunteer for the research study. Include provisions for prorating payment. See payment considerations

Subjects will be paid [redacted] for completing all aspects of the protocol, not including screening. This will be broken down as: [redacted] per week of study (5 weeks = [redacted]) + [redacted] completion incentive. This is payable after completion of the individual's study. Individuals who complete only part of the study will receive a prorated amount, not including the completion incentive.

l) **Costs. Please explain any costs that will be charged to the participant.**

Participants will not be charged for engaging in this study.

m) **Estimate the probable duration of the entire study. Also estimate the total time per participant for: (i) screening of participant; (ii) active participation in study; (iii) analysis of participant data.**

We estimate that that study will take two years to complete subject empanelment and analysis. Screening participants will take approximately three hours each. Participants will then be active in the protocol for five weeks. Analysis of data will take approximately 6 months.

9. Risks

a) For the following categories include a scientific estimate of the frequency, severity, and reversibility of potential risks. Wherever possible, include statistical incidence of complications and the mortality rate of proposed procedures. Where there has been insufficient time to accumulate significant data on risk, a statement to this effect should be included. (In describing these risks in the consent form to the participant it is helpful to use comparisons which are meaningful to persons unfamiliar with medical terminology.)

The risks of the Investigational devices.

None

The risks of the Investigational drugs. Information about risks can often be found in the Investigator's brochure.

None

The risks of the Commercially available drugs, reagents or chemicals. Information about risks can often be found in the package insert.

There is a risk of an unknown side-effect from taking this FDA-approved medication. The most common side-effect is sleepiness. As such, we require that subjects only take the medication when they have at least 7 hours of time to sleep.

The risks of the Procedures to be performed. Include all investigational, non-investigational and non-invasive procedures (e.g., surgery, blood draws, treadmill tests).

There is the possibility of a small scar to form at the site of venipuncture. This risk is increased in individuals with darker skin pigmentation.

The risks of the Radioisotopes/radiation-producing machines (e.g., X-rays, CT scans, fluoroscopy) and associated risks.

None

The risks of the Physical well-being.

The wrist worn activity monitors may cause a rash (contact dermititis) on the wrist underneath where the device makes contact with the skin. This risk is similar to wearing a wrist watch. If this occurs, we will recommend that participants move the watch and use skin cream on the rash.

The risks of the Psychological well-being.

There is a possibility of increased depressive symptoms following use of this medication. We will monitor this at the end of the first week of drug use. There is always a risk when completing questionnaires that participants will learn something about themselves that negative affects their psychological well-being.

The risks of the Economic well-being.

There is no likely risk to economic well-being.

The risks of the Social well-being.

There is no likely risk to social well-being.

Overall evaluation of Risk.

Low - innocuous procedures such as phlebotomy, urine or stool collection, no therapeutic agent, or safe therapeutic agent such as the use of an FDA approved drug or device.

b) If you are conducting international research, describe the qualifications/preparations that enable you to both estimate and minimize risks to participants. Provide an explanation as to why the research must be completed at this location and complete the [LINKFORINTERNATIONALREASEARCHFORM] International Research Form. If not applicable, enter N/A.

n/a

c) Describe the planned procedures for protecting against and minimizing all potential risks. Include the means for monitoring to detect hazards to the participant (and/or to a potential fetus if applicable). Include steps to minimize risks to the confidentiality of identifiable information.

Two weeks after initiating the baseline portion of the study, participants will return to the lab and be monitored for risk exposure. One week after initiating medication, subjects will again return to the lab and be monitored for risk exposure.

d) Explain the point at which the experiment will terminate. If appropriate, include the standards for the termination of the participation of the individual participant Also discuss plans for ensuring necessary medical or professional intervention in the event of adverse effects to the participants.

The experiment will terminate at the end of the three weeks of drug administration. In case of an adverse event during the protocol, Drs. Holty and Sullivan will be able to advise a proper course of action.

e) Data Safety and Monitoring Plan (DSMP). See guidance on Data Safety and Monitoring.

A Data and Safety Monitoring Plan (DSMP) is required for studies that present Medium or High risk to participants. (See Overall Evaluation of Risk above). If Low Risk, a DSMP may not be necessary. Multi-site Phase III clinical trials funded by NIH require the DSM Plan to have a Data Safety Monitoring Board or Committee (DSMC or DSMB). The FDA recommends that all multi-site clinical trials that involve interventions that have potential for greater than minimal risk to study participants also have a DSMB or DSMC.

The role of the DSMC or DSMB is to ensure the safety of participants by analyzing pooled data from all sites, and to oversee the validity and integrity of the data. Depending on the degree of risk and the complexity of the protocol, monitoring may be performed by an independent committee, a board (DSMC/DSMB), a sponsor's Data Safety Committee (DSC), a Medical Monitor, a sponsor's safety officer, or by the Protocol Director (PD).

Describe the following:

What type of data and/or events will be reviewed under the monitoring plan, e.g. adverse events, protocol deviations, aggregate data?

Adverse events, consent forms, and protocol deviations will be monitored.

Identify who will be responsible for Data and Safety Monitoring for this study, e.g. Stanford Cancer Institute DSMC, an independent monitoring committee, the sponsor, Stanford investigators independent of the study, the PD, or other person(s).

The PD in collaboration with Drs. Holty and Sullivan will be the responsible entity.

Provide the scope and composition of the monitoring board, committee, or safety monitor, e.g., information about each member's relevant experience or area of expertise. If the Monitor is the

Stanford Cancer Center DSMC or the PD, enter N/A.

In addition to the PD, Drs. Holty and Sullivan will consult on all medically-related issues. Both are board-certified sleep clinicians.

Confirm that you will report Serious Adverse Events (SAEs), Suspected Unexpected Serious Adverse Reactions (SUSARs), or Unanticipated Problems (UPs) to the person or committee monitoring the study in accordance with Sponsor requirements and FDA regulations.

SAE, SUSAR, and UP will each be reported to the IRB and Merck (sponsor) in accordance with the Sponsor's requirements and FDA regulations.

If applicable, how frequently will the Monitoring Committee meet? Will the Monitoring Committee provide written recommendations about continuing the study to the Sponsor and IRB?

Dr. Zeitzer will monitor all data continuously and will meet with the other members of the Monitoring Committee as needed.

Specify triggers or stopping rules that will dictate when the study will end, or when some action is required. If you specified this in Section 2g [Study Endpoints], earlier in this application enter 'See 2g'.

Any SAE or SUSAR will result in the immediate termination of the study.

Indicate to whom the data and safety monitoring person, board, or committee will disseminate the outcome of the review(s), e.g., to the IRB, the study sponsor, the investigator, or other officials, as appropriate.

The PD will disseminate the outcomes of all discussion to the IRB, study sponsor, and FDA, as appropriate.

Select One:

	The Protocol Director will be the only monitoring entity for this study.
Y	This protocol will utilize a board, committee, or safety monitor as identified in question #2 above.

10. Benefits

a) **Describe the potential benefit(s) to be gained by the participants or by the acquisition of important knowledge which may benefit future participants, etc.**

Participants may find the medication helpful for treating their shift work sleep disorder and would be able to speak with their primary care physician about the appropriateness of a prescription. This study would increase our knowledge about how to appropriately treat shift work sleep disorders.

11. Privacy and Confidentiality

Privacy Protections

a) **Describe how the conditions under which interactions will occur are adequate to protect the privacy interests of participants (e.g., privacy of physical setting for interviews or data collection, protections for follow-up interactions such as telephone, email and mail communications).**

Direct interactions will occur in a private room at VAPAHCS as well as at the Clinical Studies Unit at VAPAHCS. We will primarily use the telephone to communicate issues of scheduling with the participants.

Confidentiality Protections

b) Specify PHI (Protected Health Information). PHI is health information linked to HIPAA identifiers (see above). List BOTH health information AND HIPAA identifiers. If you are using STARR, use the Data Privacy Attestation to ensure that your request will match your IRB-approved protocol. Be consistent with information entered in section 15a.

We will obtain the following PHI:
Name
Social Security number
Telephone number
Local address
Birth date
Electronic mail addresses

c) You are required to comply with University Policy that states that ALL electronic devices: computers (laptops and desktops; OFFICE or HOME); smart phones; tablets; external hard disks, USB drives, etc. that may hold identifiable participant data will be password protected, backed up, and encrypted. See http://med.stanford.edu/datasecurity/ for more information on the Data Security Policy and links to encrypt your devices.

Provide any additional information on ALL data security measures you are taking. You must use secure databases such as https://researchcompliance.stanford.edu/panels/hs/redcap RedCap. If you are unsure of the security of the system, check with your Department IT representative. Please see http://med.stanford.edu/irt/security/ for more information on IRT Information Security Services and http://www.stanford.edu/group/security/securecomputing/mobile_devices.html for more information for securing mobile computing devices. Additionally,any PHI data on paper must be secured in an locked environment.

By checking this box, You affirm the aforementioned. Y

PHI will be stored on paper in a locked cabinet in a locked room in a locked wing of Building 4 at VAPAHCS. Deidentified data will be stored on encrypted Stanford desktop computers backed up with Crash Plan Pro.

d) Describe how data or specimens will be labeled (e.g. name, medical record number, study number, linked coding system) or de-identified. If you are de-identifying data or specimens, who will be responsible for the de-identification? If x-rays or other digital images are used, explain how and by whom the images will be de-identified.

All data are de-identified. The subject coordinator assigns a sequential random code (e.g., SUV001) each participant once the participant signs a consent form.

e) Indicate who will have access to the data or specimens (e.g., research team, sponsors, consultants) and describe levels of access control (e.g., restricted access for certain persons or groups, access to linked data or specimens).

All members of the research team will have access to all data.

f) If data or specimens will be coded, describe the method in which they will be coded so that study participants' identities cannot be readily ascertained from the code.

Our lab assigns a random code with the first part indicating the study in which the individual is participating and the second the sequential number of the participant.

g) If data or specimens will be coded, indicate who will maintain the key to the code and describe how it will be protected against unauthorized access.

The key to the code is kept on paper in a locked file cabinet in a locked room in a locked wing of Building 4 at VAPAHCS. All team members have access to this information.

h) If you will be sharing data with others, describe how data will be transferred (e.g., courier, mail) or transmitted (e.g., file transfer software, file sharing, email). If transmitted via electronic networks, describe how you will secure the data while in transit.See http://www.stanford.edu/group/security/securecomputing/

Title : A double-blind, placebo-controlled, randomized, parallel design study to examine the effectiveness of 10 mg suvorexant in improving daytime sleep in shift workers

Approval Period: **Draft**

http://www.stanford.edu/group/security/securecomputing/. Additionally, if you will be using or sharing PHI see https://uit.stanford.edu/security/hipaa https://uit.stanford.edu/security/hipaa.

n/a

i) **How will you educate research staff to ensure they take appropriate measures to protect the privacy of participants and the confidentiality of data or specimens collected (e.g. conscious of oral and written communications, conducting insurance billing, and maintaining paper and electronic data)?**

The PI is responsible for ensure that all team members are up-to-date on all privacy trainings (HIPAA, CITI).

12. Potential Conflict of Interest

Investigators are required to disclose any financial interests that reasonably appear to be related to this protocol.

Financial Interest Tasks

Investigators	Role	Email	Has Financial Interest?	Date Financial Interest Answered	Date OPACS Disclosure Submitted	Date OPACS Review Completed
Jamie Marc Zeitzer	PD	jzeitzer@stanford.edu	N	06/26/2015	N/A	N/A
Shannon Sullivan	OP	shannon.s.sullivan@stanford.edu	N	07/03/2015	N/A	N/A

13. Consent Background

13. 1 Consent — Consent for all 070215

Check if VA related Y

a) **Describe the informed consent process. Include the following.**

i) **Who is obtaining consent? (The person obtaining consent must be knowledgeable about the study.)**
ii) **When and where will consent be obtained?**
iii) **How much time will be devoted to consent discussion?**
iv) **Will these periods provide sufficient opportunity for the participant to consider whether or not to participate and sign the written consent?**
v) **What steps are you taking to minimize the possibility of coercion and undue influence?**
vi) **If consent relates to children and if you have a reason for only one parent signing, provide that rationale for IRB consideration.**

i) A study team member will obtain consent. ii) Consent will be obtained in a private room at the Palo Alto VA [redacted]. iii) Given the complexity of the protocol, we anticipate that the consent process will take approximately 60 minutes. iv) We believe that this is sufficient time for the subject to initially consider participating in the study. Subjects will also be reminded of their right to withdraw consent at any time. v) There is no time pressure on the start of enrollment of the subjects and no time pressure will be placed on the consent process. We also believe that the remuneration being offered in this study is appropriate for the amount of subject involvement being requested. vi) Not applicable

b) What is the Procedure to assess understanding of the information contained in the consent? How will the information be provided to participants if they do not understand English or if they have a hearing impairment? See HRPP Chapter12.2 for guidance.

All subjects will need to be able to comprehend English and have normal hearing. One-on-one interviewing will ensure that potential participants understand what they are being asked to do.

c) What steps are you taking to determine that potential participants are competent to participate in the decision-making process? If your study may enroll adults who are unable to consent, describe (i) how you will assess the capacity to consent, (ii) what provisions will be taken if the participant regains the capacity to consent,(iii) who will be used as a legally authorized representative, and (iv) what provisions will be made for the assent of the participant.

We will not be enrolling any decisionally impaired individuals in this study.

Additional VA questions:

i) List the people to whom you have formally delegated responsibility to obtain informed consent, and state whether they have the appropriate training to perform this activity.

Dr. Jon-Erik Holty and Ms. Yvonne Quevedo have been delegated responsibility to obtain informed consent. They are both experienced at such and are properly trained to review consent forms with subjects.

ii) Will legally effective informed consent be obtained from the participant or the participant's legally authorized representative (LAR) or both? If LAR, is it clear who can serve as LAR?

LAR will not be used.

iii) Will the circumstances of the consent process minimize the possibility of coercion or undue influence and provide the prospective participant or their representative sufficient opportunity to consider whether to participate?

The circumstances of the consent process will minimize the possibility of coercion or undue influence and provide the prospective participant or their representative sufficient opportunity to consider whether to participate.

iv) Will the circumstances of the consent process minimize the possibility of coercion or undue influence?

Discussion of the consent form will occur in a private room and able time for questions will be made available. Subjects will make the appointment for the consenting process on their own time and we do not foresee that the subject will feel, either perceived or real, any undue influence.

v) Will the information being communicated to the participant or the representative during the consent process exclude any exculpatory language through which the participant or the representative is made to waive or appear to waive the participant's legal rights, or release or appear to release the investigator, the sponsor, the institution, or its agent from liability for negligence (e.g. I give up any property rights I may have in bodily fluids or tissue samples obtained in the course of the research)?

No exculpatory language is used in the consent form.

vi) Please confirm the following:

a. A witness to the participant's signature or the participant's legally authorized representative's signature will sign and date the consent document.

b. If the sponsor or the IRB requires a witness to the consenting process in addition to the witness to the participant's signature and if the same person is needed to serve both capacities, a note to that effect is placed under the witness's signature line.

c. A copy of the signed and dated consent document will be given to the person signing the consent document.

d. The consent form is on the VA Form 10-1086.

14. Assent Background (less than 18 years of age)

15. HIPAA Background

15. 1 Waiver of Authorization for Recruitment — limited waiver for phone screen

a) Describe the protected health information (PHI) needed to conduct screening or recruitment. PHI is health information linked to HIPAA identifiers. List BOTH health information AND HIPAA identifiers. If you are using STARR, use the Data Privacy Attestation to ensure that your request will match your IRB-approved protocol.

PHI collected during the phone screen includes the potential participant's name and contact information (phone number and address).

b) Please Answer:

Y **Do you certify that the use or disclosure of protected health information involves no more than a minimal risk to the privacy of individuals?**

Y **Do you certify that the research could not practically be conducted with out the waiver?**

Y **Do you certify that you have adequate written assurances that the protected health information will not be reused or disclosed to any other person or entity, except as required by law, for authorized oversight of the research project, or for other research for which the use or disclosure of protected health information would be permitted?**

Y **Do you certify that the research could not practically be conducted with out access to and use of the protected health information?**

c) Please describe an adequate plan to protect any identifiers from improper use and disclosure.

Phone screens will be kept in a binder separate from other study information. The binder will be kept in a locked drawer in a locked room.

d) Please describe an adequate plan to destroy the identifiers at the earliest opportunity consistent with conduct of the research, unless there is a health or research justification for retaining the identifiers or such retention is otherwise required by law.

Once the study has been completed, PHI information will be destroyed.

16. Attachments

Attachment Name	Attached Date	Attached By	Submitted Date
belsomra package insert	06/26/2015	jzeitzer	
Bergen Shift Work Sleep Questionnaire	06/26/2015	jzeitzer	
RLS question	06/26/2015	jzeitzer	
CES-D subject form	06/26/2015	jzeitzer	
Morningness Eveningness Questionnaire	06/26/2015	jzeitzer	
Duke Sleep interview - CRSD module	06/26/2015	jzeitzer	
Insomnia Severity Index (night sleep)	06/26/2015	jzeitzer	
Insomnia Severity Index (day sleep)	06/26/2015	jzeitzer	

CGI-S and -I	06/26/2015	jzeitzer	
Fosq98 short version	06/29/2015	jzeitzer	
demographics-r063015	06/30/2015	jzeitzer	
Modified CSD_Core Day Sleep	07/01/2015	jzeitzer	
Modified CSD_Core Night Sleep	07/01/2015	jzeitzer	
VARQs_APP1m	07/02/2015	jzeitzer	

Obligations

The Protocol Director agrees to:

- Adhere to principles of sound scientific research designed to yield valid results
- Conduct the study according to the protocol approved by the IRB
- Be appropriately qualified to conduct the research and be trained in Human Research protection, ethical principles, regulations, policies and procedures
- Ensure all Stanford research personnel are adequately trained and supervised
- Ensure that the rights and welfare of participants are protected including privacy and confidentiality of data
- Ensure that, when de-identified materials are obtained for research purposes, no attempt will be made to re-identify them.
- Disclose to the appropriate entities any potential conflict of interest
- Report promptly any new information, modification, or unanticipated problems that raise risks to participants or others
- Apply relevant professional standards.

Any change in the research protocol must be submitted to the IRB for review prior to the implementation of such change. Any complications in participants or evidence of increase in the original estimate of risk should be reported at once to the IRB before continuing with the project. Inasmuch as the Institutional Review Board (IRB) includes faculty, staff, legal counsel, public members, and students, protocols should be written in language that can be understood by all Panel members. The investigators must inform the participants of any significant new knowledge obtained during the course of the research.

IRB approval of any project is for a maximum period of one year. For continuing projects and activities, it is the responsibility of the investigator(s) to resubmit the project to the IRB for review and re-approval prior to the end of the approval period. A Notice to Renew Protocol is sent to the Protocol Director 7 weeks prior to the expiration date of the protocol.

Department Chair must approve faculty and staff research that is not part of a sponsored project. VA applicants must have Division Chief or Ward Supervisor approval. E-mail the Department Chair approval to IRBCoordinator@lists.stanford.edu.

All data including signed consent form documents must be retained for a minimum of three years past the completion of the research. Additional requirements may be imposed by your funding agency, your department, or other entities. (Policy on Retention of and Access to Research Data, Research Policy Handbook,

Title : A double-blind, placebo-controlled, randomized, parallel design study to examine the effectiveness of 10 mg suvorexant in improving daytime sleep in shift workers

Approval Period: **Draft**

http://doresearch.stanford.edu/policies/research-policy-handbook/conduct-research/retention-and-access-research-data)

PLEASE NOTE: List all items (verbatim) that you want to be reflected in your approval letter (e.g., Amendment, Investigator's Brochure, consent form(s), advertisement, etc.) in the box below. Include number and date when appropriate.

Y By checking this box, I verify that I, as the Protocol Director (PD) responsible for this research protocol, have read and agree to abide by the above obligations, or that I have been delegated authority by the PD to certify that the PD has read and agrees to abide by the above obligations.

**2.7 Statistical Analysis and Sample Size Justification**	The PI, Dr. Jamie Zeitzer, will be responsible for analyzing all study data. The blind will be held at the local site by the VAPAHCS research pharmacist. Once all data has been cleaned and locked, Dr. Zeitzer will be unblinded to conduct the final analyses.<br><br><u>Variables/Time Points of Interest</u><br>Objective and subjective sleep variables (total sleep time, sleep efficiency, wake after sleep onset, sleep quality) will be collected continuously during the 2-week baseline and the three-week intervention. Subjective alertness assessment will also be collected continuously over the five-week protocol. All of these will be used in the study analyses.<br><br><u>Statistical Methods</u><br>The purpose of this study is to test the hypothesis that ingestion of 10 mg of suvorexant 30 minutes prior to daytime sleep initiation in individuals working overnight shifts will significantly improve both objective (total sleep time, sleep efficiency, wake after sleep onset) and subjective (sleep quality) measures of sleep. A secondary goal is to test the hypothesis that ingestion of 10 mg of suvorexant 30 minutes prior to daytime sleep initiation will (1) increase subjective measures of alertness during the night shift and (2) decrease total sleep time during nocturnal sleep episodes on off-days. To accommodate the nested nature of the data (days nested within persons), analyses will be conducted in a multilevel modeling framework (19). Specifically, the total sleep time (TST), as an example, will be modeled as: $TST\#_{it} = \gamma_{00} + \gamma_{01}*group_i + u_{0i} + e_{it}$,, where the *TST* obtained above for person *i* on day *t*, $TST\#_{it}$, are modeled separately as a function of between-person and within-person differences. Specifically, γ_{00}, indicates the expected *TST* score for a prototypical participant on a prototypical day (the reference category); γ_{01} captures between-group differences in sleep (placebo group vs. drug group); and u_{0i} and e_{it} capture additional between-person and occasion-specific differences not otherwise explained. All models will be fit using SAS 9.4 (proc mixed) with restricted maximum likelihood estimation. Effect size will be evaluated with respect to proportional reduction in unexplained variance from an informative baseline model (*pseudo*-R^2, see (19)).<br><br><u>Exploratory measures:</u><br>While the primary measures in this study will examine *prospective* changes in sleep, we will also collect data that will examine *retrospective* changes in sleep. These include examining changes in subjective measures of sleep quality that are captured

	in the BSWSQ, ISI, and FOSQ-10 questionnaires. Changes on these questionnaires will be examined with repeated measure ANOVA. Another exploratory measure to be analyzed in the same manner will be a change in depressive symptom severity as captured on the CES-D. A final exploratory measure will be to examine the change in the number of caffeinated beverages during work shifts. Given that there is tremendous variability in the caffeine content of caffeinated beverages, within subject changes will be examined with multilevel modeling.<br><br><u>Power/Sample Size:</u><br>For estimation of sample size, we have reduced our aim to the single, most important outcome measure - will sleep efficiency improve in the drug condition - and reduced the statistical testing to a *t*-test. In previously published work (20), a variance of 3.62% - 3.87% was observed following use of 10 mg of suvorexant or placebo. For purposes of this sample size estimation, we can estimate σ = 5.55% (average variance * 150%, with the increase due to our use of actigraphy instead of polysomnography as a reporter). Setting α = 0.05 and power = 80%, we would be able to detect a difference of 5.2% in sleep efficiency (i.e., that which was observed in a previous trial of 10 mg suvorexant in those with insomnia (21)) with 19 participants in each group (n=38 total).

1
