# Supplementary material for: Effect of Suvorexant vs Placebo on Total Daytime Sleep Hours in Shift Workers: A Randomized Clinical Trial
Source: JAMA Netw Open. 2020 Jun 2;3(6):e206614. doi: 10.1001/jamanetworkopen.2020.6614 (PMC7267849; doi:10.1001/jamanetworkopen.2020.6614)
Supplement: Supplement 2. — Data Sharing Statement [file jamanetwopen-3-e206614-s002.pdf]

Data Sharing Statement

Zeitzer. Effect of Suvorexant vs Placebo on Total Daytime Sleep Hours in Shift Workers. *JAMA Netw Open*. Published June 02, 2020. 10.1001/jamanetworkopen.2020.6614

Data
Data available: Yes
Data types: Deidentified participant data, Data dictionary
How to access data: jzeitzer@stanford.edu
When available: With publication

Supporting Documents
Document types: None

Additional Information
Who can access the data: researchers whose proposed use of the data has been approved
Types of analyses: for any purpose
Mechanisms of data availability: signed data access agreement
